# *In Vitro* Inhibition of Histamine Release Behavior of Cetirizine Intercalated into Zn/Al- and Mg/Al-Layered Double Hydroxides

**DOI:** 10.3390/ijms13055899

**Published:** 2012-05-16

**Authors:** Samer Hasan Hussein-Al-Ali, Mothanna Al-Qubaisi, Mohd Zobir Hussein, Maznah Ismail, Zulkarnain Zainal, Muhammad Nazrul Hakim

**Affiliations:** 1 Department of Chemistry, Faculty of Science, Universiti Putra Malaysia, Selangor 43400, Malaysia; E-Mails: sameralali72@yahoo.com (S.H.H.-A.-A.); zulkar@putra.upm.edu.my (Z.Z.); 2 Laboratory of Molecular Biomedicine, Institute of Bioscience, Universiti Putra Malaysia, Selangor 43400, Malaysia; E-Mail: mothanna_alqubaisi@yahoo.com; 3 Advanced Materials and Nanotechnology Laboratory, Institute of Advanced Technology (ITMA), Universiti Putra Malaysia, Selangor 43400, Malaysia; 4 Department of Nutrition and Dietetics, Faculty of Medicine and Health Science, Universiti Putra Malaysia, Selangor 43400, Malaysia; E-Mail: maznah@medic.upm.edu.my; 5 Department of Biomedical Science, Faculty of Medicine and Health Science, Universiti Putra Malaysia, Selangor 43400, Malaysia; E-Mail: nazrul.hakim@gmail.com

**Keywords:** (Zn/Al, Mg/Al)-layered double hydroxides, cetirizine, nanocomposite, controlled release, RBL2H3 cell, human Chang liver cells

## Abstract

The intercalation of cetirizine into two types of layered double hydroxides, Zn/Al and Mg/Al, has been investigated by the ion exchange method to form CTZAN and CTMAN nanocomposites, respectively. The basal spacing of the nanocomposites were expanded to 31.9 Å for CTZAN and 31.2 Å for CTMAN, suggesting that cetirizine anion was intercalated into Layered double hydroxides (LDHs) and arranged in a tilted bilayer fashion. A Fourier transform infrared spectroscopy (FTIR) study supported the formation of both the nanocomposites, and the intercalated cetirizine is thermally more stable than its counterpart in free state. The loading of cetirizine in the nanocomposite was estimated to be about 57.2% for CTZAN and 60.7% CTMAN. The cetirizine release from the nanocomposites show sustained release manner and the release rate of cetirizine from CTZAN and CTMAN nanocomposites at pH 7.4 is remarkably lower than that at pH 4.8, presumably due to the different release mechanism. The inhibition of histamine release from RBL2H3 cells by the free cetirizine is higher than the intercalated cetirizine both in CTZAN and CTMAN nanocomposites. The viability in human Chang liver cells at 1000 μg/mL for CTZAN and CTMAN nanocomposites are 74.5 and 91.9%, respectively.

## 1. Introduction

Pharmaceutical science usually suffers from short time release of the active compounds in the body to maintain the therapeutic window. Sustained release of drug for more effective delivery systems have received intense attention in recent years from the pharmaceutical industry due to the advantages they offer over conventional forms [1]. Layered double hydroxide (LDH) is one of the carriers which many offer, the benefits of which include prolongation of drug action, a decrease or elimination of side effects and enhanced efficiency of drug delivery. LDH has 2-D, positively-charged layers which are produced from the isomorphous substitution of magnesium cations by trivalent cations in a brucite structure. The brucite structure of the magnesium hydroxide, Mg(OH)_2_ is composed of magnesium cations which are located as centered to six octahedrally hydroxyl groups. Each hydroxide is bonded to three magnesium atoms, resulting in neutral layer structure. To neutralize the positive charge of LDH structure, counter anions are intercalated between the interlayers which give the hydrotalcite-like structure with the general formula [M_1−x_^2+^ M_x_^3+^ (OH)_2_]*^x^*^+^ (A^m−^)*_x_*_/_*_m_*. *n*H_2_O, where M^II^ are divalent cations, M^III^ are trivalent cations and A^m−^ is an exchangeable anion with charge (m^−^) [2].

The intercalation of drugs as guest molecules into LDH can be accomplished by different methods; the two most common are coprecipitation and ion exchange [3]. Recently, many LDH compounds with intercalated drug anions have been elucidated, such as the anticancer drugs: methotrexate [4], 5-fluorouracil [5] and podophyllotoxin [6]; the anti inflammatory drugs: fenbufen, diclofenac, ibuprofen and camptothecin [7–10]; the anti-hypertensition drugs: captopril [11], perindopril erbumine [12] and ramipril [13]; the antifungal, 5-fluorocytosie [14], and finally the antibiotic drugs, gramicidin, amphotericin B, ampicillin and nalidixic acid [15].

Antihistamine drugs prevent histamine release by displacement from the receptor. Histamine release can happen in the case of tissue injury and allergic reactions [16]. Cetirizine dihydrochloride ((2-1-piperazinyl ethoxy) acetic acid) is one of a second-generation of antihistamines which can inhibit histamine release by blocking the H1 receptor.

The intercalation of antihistamine into LDH is interesting and, to date, cannot be found in the open literature. Therefore, in this work, we discuss the intercalation of cetirizine into Zn/Al- and Mg/Al-LDH. We will concentrate on the spatial orientation of cetirizine moiety between the layers and its release properties. In addition, we also report here the study of the effect of the resulting nanocomposites on the histamine release from RBL2H3 cells as well as their LDH counterparts, Zn/Al- and Mg/Al-LDH.

## 2. Results and Discussion

### 2.1. Powder X-Ray Diffraction

**Figure 1:**
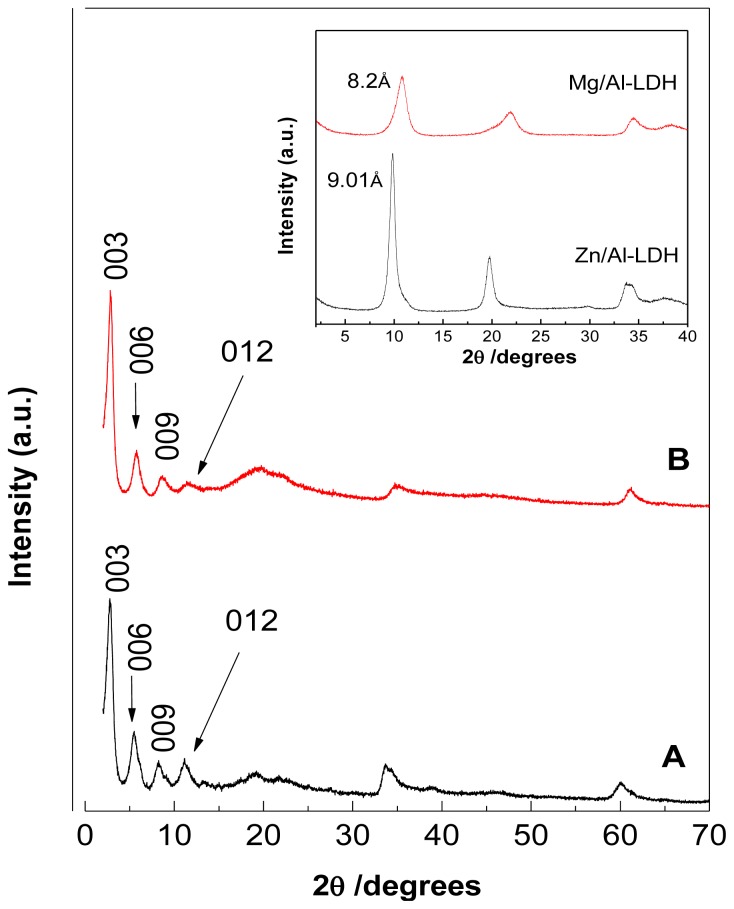
Powder X-ray diffraction patterns of (**A**) CTZAN and (**B**) CTMAN nanocomposites. Inset: XRD diffraction for Zn/Al-LDH and Mg/Al-LDH.

XRD patterns of the nanocomposites demonstrate successful intercalation of cetirizine into both fresh Zn/Al- and Mg/Al-LDH hosts (Figure 1A,B, respectively). During ion-exchange nitrate anions, the *d*- spacing of inorganic layers, Zn/Al- and Mg/Al-LDH, which are 9.01 and 8.2 Å, respectively was expanded for the host cetirizine anions. This expansion is reflected by the d-spacing values that were calculated from the mean values of the first, second, third and fourth order peaks of the XRD patterns, which are 31.9, 15.93, 10.64 and 7.88 Å, respectively for CTZAN, and 31.15, 15.33, 10.15 and 7.66 Å, respectively for CTMAN. As a result, a basal spacing value of 31.8 Å for CTZAN and 30.8 Å for CTMAN was obtained. In the case of CTZAN, the XRD patterns show one peak at 2*θ* 26° (*d* = 9.54 Å), which is overlapping with a peak at 11.22°. This result indicates the presence of some un-reacted Zn/Al-NO_3_-LDH remains in the sample. Previous studies have indicated that it is difficult to exchange nitrate in LDHs with the incoming anions, due to the parallel orientation of nitrate ions with respect to the hydroxide layers. As a result, some nitrate anions remained after the intercalation process of cetirizine into Zn/Al LDH and this explained why some un-reacted Zn/Al-NO_3_-LDH remained [17]. A slight discrepancy in the *d*_003_ value between CTZAN and CTMAN nanocomposites is probably mainly due to the content of water in the interlayer galleries and the presence of nitrate ions in the interlayer region [18].

The lattice parameters, *a* and *c* of the PMAE and PZAE nanocomposites are listed in Table 1 and were calculated using the *d*-values of 003, 006 and 009 reflections, where (*c* = 1/3(3*d*_003_ + 6*d*_006_ + 9*d*_009_)), and 110 reflection for parameter *a*, where (*a* = 2*d*_110_). Table 1 shows that the *c* value for CTZAN and CTMAN is 95.5 Å and 92.4 Å, respectively, which suggests that the cetirizine anions should be arranged in the interlayer space in a similar fashion, and that the small differences between them can be related to the conditions of preparation and/or more probably, the water content, as mentioned earlier.

### 2.2. Molecular Structure and Spatial Orientation of the Intercalated Cetirizine

**Figure 2:**
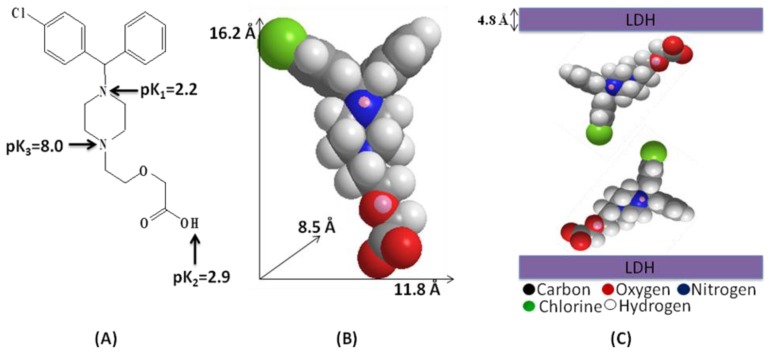
Modular structure of cetirizine (**A**), three-dimensional molecular size of cetirizine (**B**) and spatial orientation of cetirizine between LDH inorganic interlayers (**C**).

Figure 2A shows the three ionizable forms of cetirizine; a strong acidic group with pK_2_ = 2.9, a strong basic group with pK_3_ = 8.0 and a very weakly basic group with pK_1_ = 2.2 [19]. According to XRD analysis, the *d* spacing (*d*_003_) increased to 31.8 Å and 30.8 Å for CTZAN and CTMAN, respectively. Due to the value of thickness of the Zn/Al and Mg/Al-LDH layers are constant, which is 4.8 Å [20], thus, the gallery height of LDH after intercalation can be calculated by the d spacing minus the thickness of the LDH layer which is 27 Å (31.8–4.8) for CTZAN and 26 Å (30.8–4.8) for CTMAN. The long, short axis and thickness of cetirizine anion was calculated using a Chemoffice software (Cambridge, MA), and they are 16.2, 8.5 and 11.8 Å, respectively (Figure 2B). The gallery height of CTZAN and CTMAN nanocomposites are far beyond the value of the long axis. It is also slightly smaller than twice of the long axis dimension (32.4 Å). This suggests that cetirizine anions are accommodated as alternately tilted bilayer along the long axis orientation in certain angle between LDH layers, where the carboxyl anion group attaching to the upper or lower layers, respectively.

### 2.3. Fourier Transforms Infrared Spectroscopy

**Figure 3:**
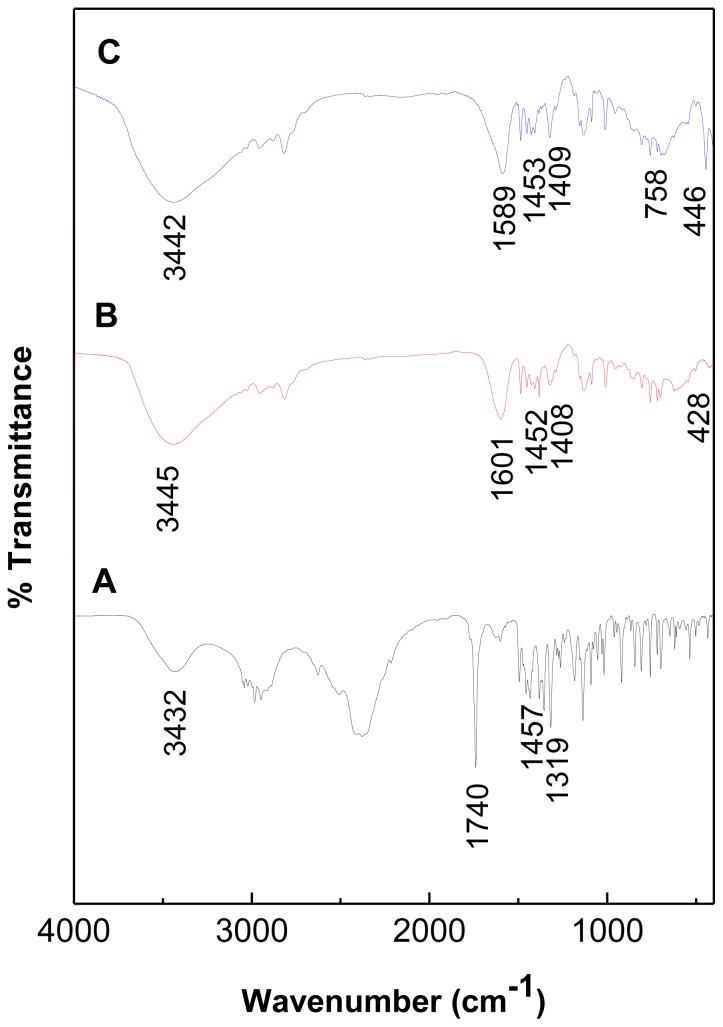
FTIR spectra of cetirizine (**A**), CTZAN (**B**) and CTMAN(**C**).

Figure 3 and Table 2 show FTIR spectra of the cetirizine, CTZAN and CTMAN nanocomposites. As shown in Figure 3A for pure cetirizine, a broad band at 3432 cm^−1^ is due to the O–H stretching vibration [21]. A band recorded at 3044–3023 cm^−1^ is due to C–H stretching of the aromatic ring. A (C=O) of the carboxylic group gives a strong band at 1740 cm^−1^. A band at 1457 cm^−1^ is due to C–Cl stretching. Mono-substitution on the benzene ring gives three different absorption bands at 1496, 1077 and 758 cm^−1^. Substitution at *para* position on the benzene ring gives a band at 1602 cm^−1^. Bending two adjacent benzene rings give bands at 846–809 cm^−1^.

The FTIR spectra of CTZAN and CTMAN nanocomposites are shown in Figure 3B,C, respectively, show characteristic bands of cetirizine. This indicates that the cetirizine anions have been intercalated into the interlayer galleries of the Zn/Al- and Mg/Al-LDH. However, some of the bands are slightly shifted in position, due to interaction between cetirizine anions and the inorganic LDH host interlayers. Table 2 shows the FTIR assignments for CTZAN and CTMAN nanocomposites. An intense bands at 1601 and 1408 cm^−1^ of the CTZAN are attributed to asymmetric and symmetric carboxylate stretching of the cetirizine anions, respectively. The CTMAN nanocomposite shows bands at 1589 and 1408 cm^−1^ for asymmetric and symmetry stretching, respectively. Peaks at 428 cm^−1^ and 446 cm^−1^ are associated with M-O stretching modes in the CTZAN and CTMAN, respectively [5,22].

### 2.4. Elemental Analysis

Elemental analysis was done for the organic and inorganic composition of CTZAN and CTMAN, as shown in Table 3. As expected, the CTZAN and CTMAN nanocomposites contained both organic and inorganic constituents. Both elemental analysis and XRD study indicates that intercalation occurred in which cetirizine was intercalated into the LDH inorganic interlayers.

Table 3 shows that the percentage loading of cetirizine in CTZAN and CTMAN nanocomposites are 57.2 and 60.7%, respectively. The C/N ratio for free cetirizine is 9.1 and this value is similar to CTMAN nanocomposite, indicating complete removal of the nitrate anion between the interlayer. In case of CTZAN, the ratio is less than 9.1; it is about 8.8, which is due to the presence of un-reacted ZnAl-NO_3_. This result was confirmed by the presence of a very low intensity peak at 2*θ* 9.26° (*d* = 9.54 Å) in the XRD pattern. From the elemental chemical analysis and thermogravimetric studies, the empirical formula was derived as shown in Table 3 for both CTZAN and CTMAN nanocomposites.

### 2.5. Thermal Study

**Figure 4:**
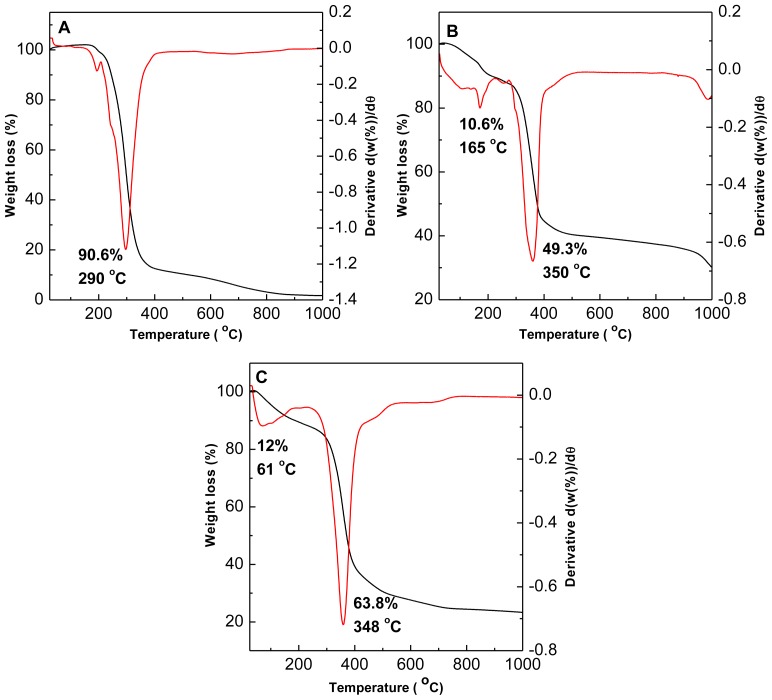
TGA/DTG thermograms of (**A**) cetirizine, (**B**) CTZAN and (**C**) CTMAN.

TGA/DTG thermogravimetric analyses obtained for cetirizine hydrochloric acid, CTZAN and CTMAN nanocomposites are reported in Figure 4. For free cetirizine (Figure 4A), the thermal behavior shows that the temperature maxima is at 290 °C with weight loss of 90.6% compared to 350 °C for the CTZAN and 348 °C for the CTMAN. This indicates that cetirizine encapsulated into the inorganic interlamellae is thermally more stable than their counter part in the free form. It is worth mentioning that very small weight loss in Figure 4A started at 159 °C to 210 °C is presumably due to the evolution of hydrochloric acid [24]; the weight loss for cetirizine is stopped at 900 °C due to the vaporization of the remaining cetirizine material.

For CTZAN nanocomposite, two stages of weight loss were observed in Figure 4B. The weight losses occur at temperature maxima of 165 and 350 °C with weight losses of 10.6% and 49.3%, respectively. The first stage of weight loss is attributed to the removal of surface physisorbed and intercalated water molecules, followed by the second stage of weight loss, which is due to the decomposition of interlayer cetirizine anion and dehydroxylation of the hydroxyl layer at 350 °C with 49.3% weight loss. Similar to free cetirizine, the weight loss in CTZAN nanocomposite stopped at 999 °C with 10 % weight loss from 506 to 999 °C.

Figure 4C shows the thermal behavior of CTMAN nanocomposite, with two major stages of weight loss process occurring at the temperature maxima of 61 and 348 °C, with weight losses of 12 and 63.8%, respectively. The first weight loss corresponds to removal of water physisorbed and interlayer strongly held water molecules. The second weight loss is complete at 726 °C and corresponds to removal of hydroxyl groups from the layers and the decomposition of the cetirizine anions with 63.8% weight loss.

### 2.6. Surface Characterization

**Figure 5:**
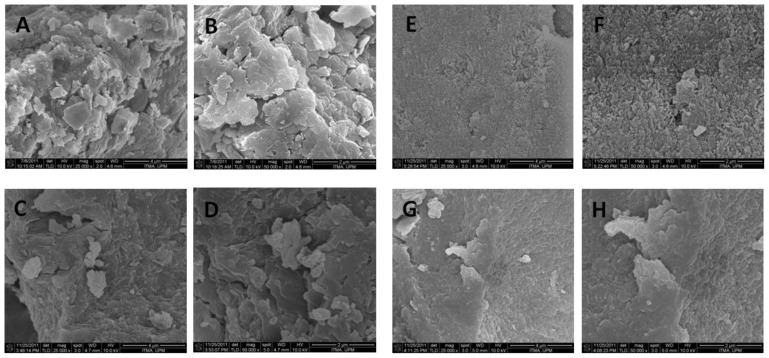
FESEM microscopic images of ZnAl-LDH (**A** and **B**), CTZAN (**C** and **D**), MgAl-LDH (**E** and **F**) and CTMAN (**G** and **H**).

The surface morphology of Zn/Al-, Mg/Al-LDH, CTZAN and CTMAN nanocomposites are shown in Figure 5. The micrographs were obtained using a FESEM (Figure 5A,C,E,G) at 25,000× and (Figure 5B,D,F,H) at 50,000× magnifications. Zn/Al-LDH and CTZAN shows non-uniform, irregular agglomerates of compact and non-porous plate-like structure (Figure 5A–D). Mg/Al-LDH exhibits a plate-like morphology (Figure 5E,F). Formation of the nanocomposite, CTMAN has resulted in a morphology change to agglomerates of compact and non-porous granular structure (Figure 5G,H).

### 2.7. Release Behavior of Cetirizine

**Figure 6:**
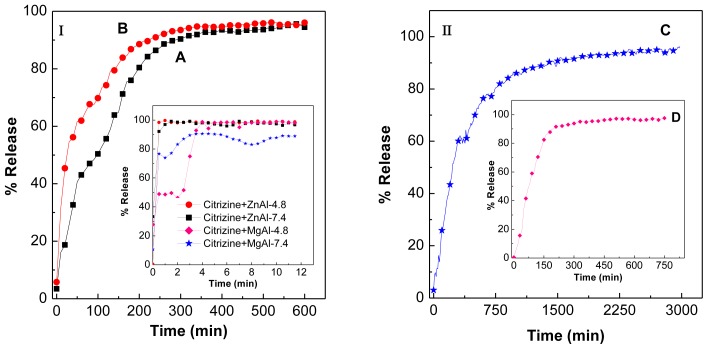
(**I**) Release profiles of cetirizine from the CTZAN nanocomposite at pH 7.4 (**A**) and pH 4.8 (**B**); and (**II**) release profiles of cetirizine from the CTMAN nanocomposite at pH 7.4 (**C**) and pH 4.8 (**D**). Inset in (**I**) shows the release profiles of cetirizine from its physical mixture of cetirizine with LDH at pH 7.4 and pH 4.8.

The release profiles of cetirizine from the CTZAN and CTMAN nanocomposites and the physical mixture of cetirizine and the pristine Zn/Al- and Mg/Al-LDH are shown in Figure 6. It can be seen from inset of Figure 6I that the physical mixture of cetirizine and pristine LDH exposed to either a pH 4.8 or 7.4 environment, cetirizine was released quickly; the release was completed within 5 min. The release rate of cetirizine from the CTZAN and CTMAN nanocomposites are obviously slower than that from the physical mixture as shown in the Figure 6I,II, indicating that both the nanocomposites are potential drug controlled release systems. This may be attributed to the electrostatic interaction between layers of LDHs and cetirizine anions intercalated into the interlayers.

In addition, the acidity of the media can also affect the release rate of cetirizine from the nanocomposites. The release rate at pH 7.4 is remarkably lower than that at pH 4.8. The percentage release of cetirizine from the CTZAN and CTMAN nanocomposites reaches about 95.6 and 96.3% within about 600 and 2980 min, respectively when exposed to the pH 7.4 environment (Figure 6A,C); compared to only about 96 and 97.8% within about 600 and 750 min at pH 4.8 (Figure 6B,D), respectively. Such differences in the release rate at pH 4.8 and 7.4 may be possibly due to the difference in mechanism for the release of cetirizine from the nanocomposites [25]. At pH 4.8, the LDH is unstable and may be dissolved, thus the release of cetirizine molecules occurs by removal of LDHs layer. At pH 7.4, the LDHs should be more stable and the release occurs through a diffusion and dissolution of LDHs.

The release rate from CTMAN is obviously very much lower than CTZAN. Completed 96.3% release was observed within 2980 min compared to 95.6% within 600 min at pH 7.4. These results are due to the charge density in which the charge density, *x* for CTMAN is 0.33, which is higher than the CTZAN, 0.23 (Table 1). Increases in the charge density will lead to the increase in the electrostatic interaction between the positively charged layers of LDH lattice and the interlayer anions that to be released.

### 2.8. Release Kinetics of Cetirizine from CTZAN and CTMAN Nanocomposites

The release behavior of cetirizine from CTZAN and CTMAN nanocomposites can be described by different kinetics models, pseudo-first order (Equation (1)) [26], pseudo-second order (Equation (2)) [27] and parabolic diffusion (Equation (3)) [28] equations;

(1) $$\text{ln }(q_{\text{e}}-q_{\text{t}})=\text{ln }q_{\text{e}}-k_{1}t$$

(2) $$t/q_{\text{t}}=1/k_{2}{q_{\text{e}}}^{2}+t/q_{\text{e}}$$

(3) $$(1-M_{\text{t}}/M_{0})/t={kt}^{-0.5}+b$$

where, *M*_0_ and *M*_t_ are the drug content remained in the LDH at release time 0 and *t*, respectively, *q*_e_ and *q*_t_ are the equilibrium release amount and the release amount at time *t*, respectively; and *k* is the corresponding release rate constant.

**Figure 7:**
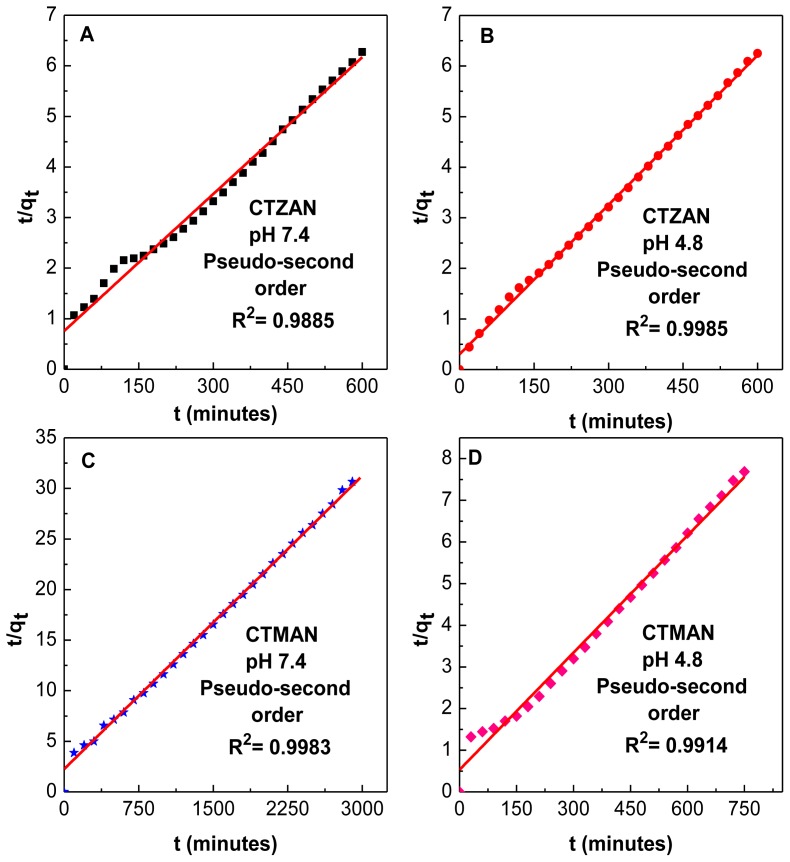
Fitting the data of cetirizine release from CTZAN (**A** and **B**) and CTMAN (**C** and **D**) into pseudo-second order kinetics model at pH 7.4 and 4.8.

With the use of the three kinetic models as mentioned earlier for the release kinetic data; it was found that the pseudo-second order model is more satisfactory for describing the release kinetic processes of cetirizine from the nanocomposites compared to the other models used in this work. Figure 7 shows the plots of *t*/*q*_t_ against *t* for pseudo-second order kinetic model, and the resulting correlation coefficient and the rate constant *k*_2_ values is given in Table 4. For the CTZAN, the correlation coefficient (*R*^2^) and *k*_2_ values are 0.9885, 1.07 × 10^−4^ and 0.9985, 3.24 × 10^−4^ at pH 7.4 and 4.8, respectively. For the CTMAN, the value is 0.9983, 4.14 × 10^−5^ and 0.9914, 1.65 × 10^−4^ at pH 7.4 and 4.8, respectively. The results of kinetic model obtained in this work are similar to the kinetic study for the release of camptothecin from Mg/Al layered double hydroxide [26] and also very similar to the intercalated perindopril erbumine into Zn/Al-layered double hydroxide [12].

### 2.9. Cytotoxicity of CTZAN and CTMAN Nanocomposites toward Human Chang Liver Cells Line

**Figure 8:**
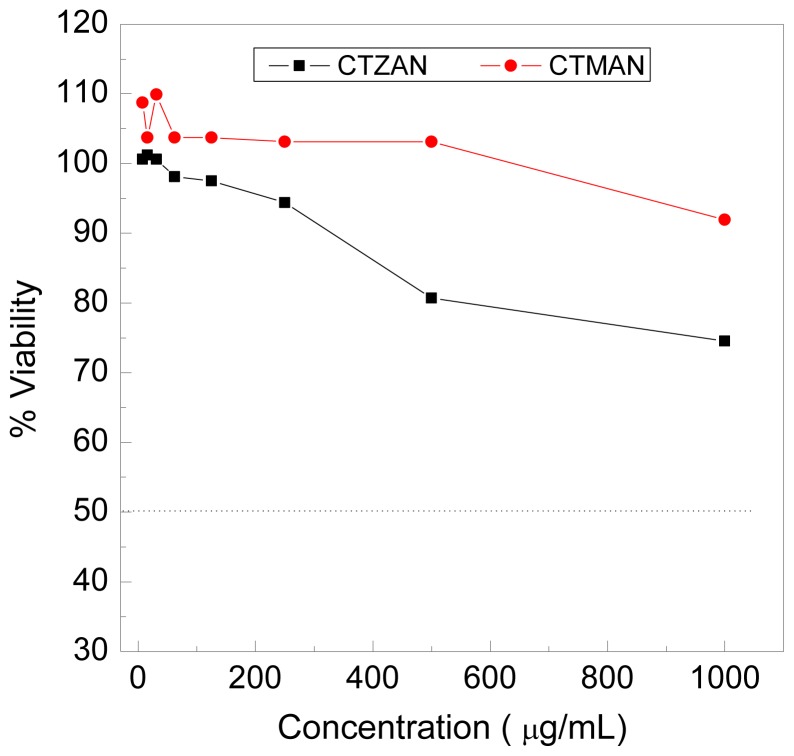
Effect of CTZAN and CTMAN nanocomposites on viability of human Chang liver cells line.

The possibility of toxic effect for CTZAN and CTMAN nanocomposites toward human Chang liver cells line was evaluated. As shown in Figure 8, no cytotoxicity effect for both nanocomposites up to 1000 μg/mL. In case of CTZAN, the nanocomposite is slightly more toxic than the CTMAN, where the viability at 1000 μg/mL for CTZAN and CTMAN nanocomposites are 74.5 and 91.9%, respectively.

### 2.10. Effect of Cetirizine, CTZAN, CTMAN, Zn/Al-NO_3_ and Mg/Al-NO_3_ on Histamine Release from RBL2H3 Cells

The histamine is formed from the histidine dehydroxylation, and it is stored in tissue mast cells and basophilic granulocytes in the blood. Histamine produces in the body in such cases, for examples; tissue injury and allergic reactions. Cetirizine is one of the second-generation antihistamines acting by displacing histamine from the H1 receptor. The increased of histamine secretion in the body may be responsible for several diseases, such as diarrhea, abdominal pain and cramping [29].

**Figure 9:**
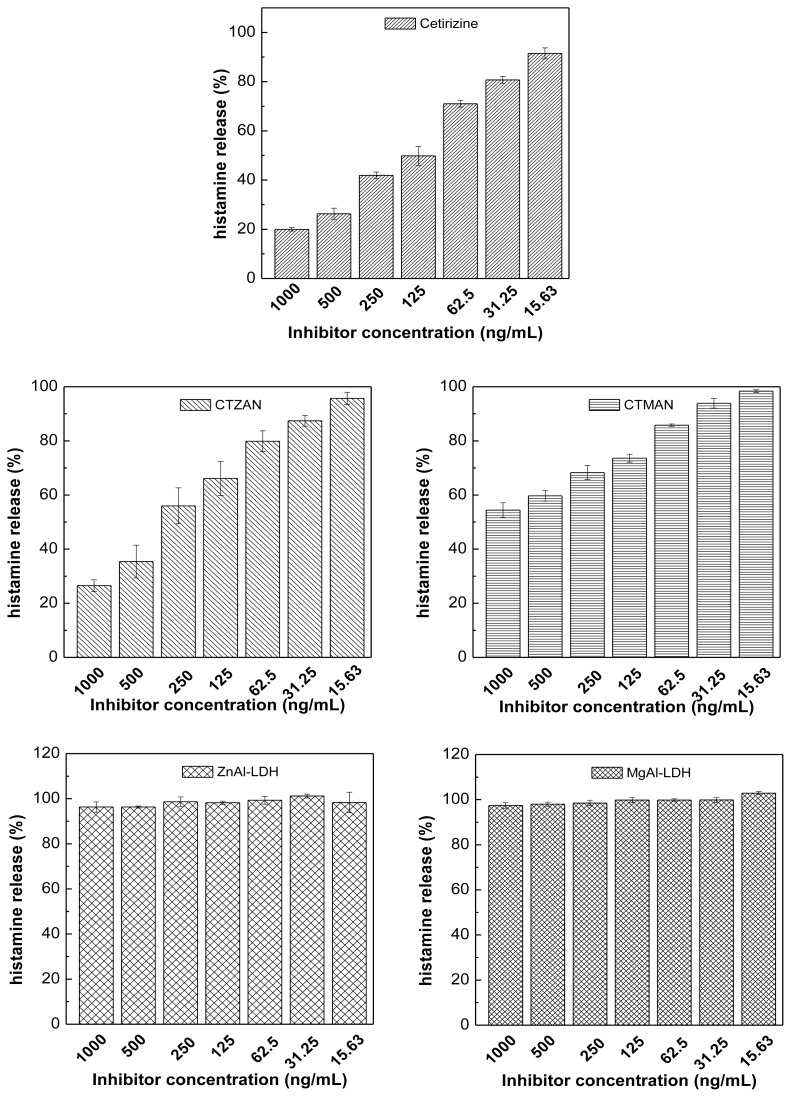
Histamine release response of RBL2H3 cells treated at different concentrations of cetirizine, CTZAN, CTMAN, Zn/Al-LDH and Mg/Al-LDH.

The activity of free cetirizine and intercalated cetirizine in CTZAN and CTMAN nanocomposites, as well as LDHs, both Zn/Al-LDH and Mg/Al-LDH are evaluated by determining their inhibitory potencies of histamine release into RBL2H3 cells (Figure 9). As shown in the figure, the effect of the components on histamine release from RBL2H3 cell was increased as the concentration of the components decreased. However, histamine release reached 20, 26.5 and 54.4% at 1000 ng/mL concentration for free cetirizine, CTZAN and CTMAN, respectively. due to the impact effect of zinc and magnesium on the histamine release [30,31], therefore, obviously Zn/Al-LDH and Mg/Al-LDH show different histamine release properties (Figure 9).

**Figure 10:**
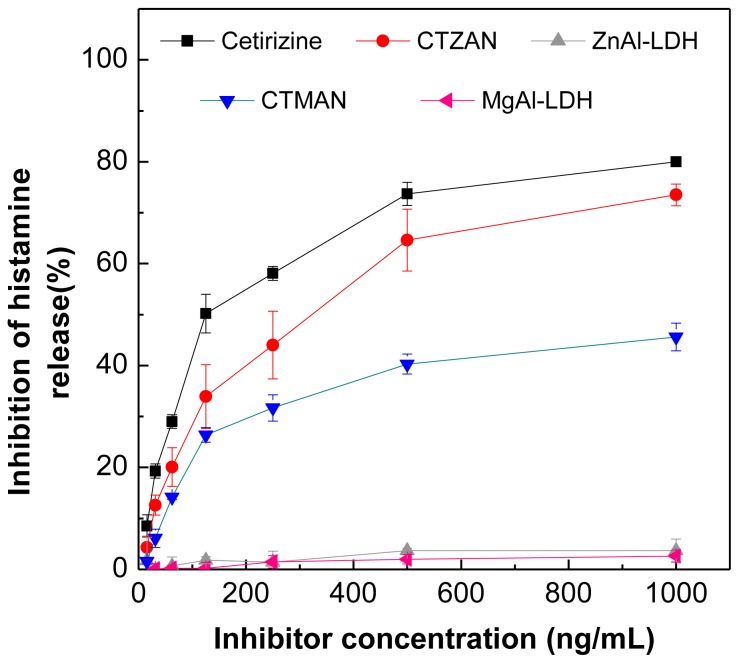
Percentage inhibition of histamine release into RBL2H3 cells at different concentrations of cetirizine, CTZAN, CTMAN, Zn/Al-LDH and Mg/Al-LDH.

The inhibition of histamine release by free cetirizine, CTZAN and CTMAN are shown in Figure 10 and Table 5. The inhibition of histamine by the free cetirizine is higher than the intercalated cetirizine into CTZAN and CTMAN nanocomposites. For example, at 1000 ng/mL the free cetirizine shows 80% inhibition compared to CTZAN and CTMAN nanocomposites which shows only 73.5% and 45.6% inhibition, respectively. This result may be due to the small amount of cetirizine made available for the RBL-2H3 cells study from CTZAN and CTMAN nanocomposites, due to their controlled release property. The cells were treated with nanocomposites for only 10 min, and during this time, the release amount of cetirizine from CTZAN and CTMAN nanocomposites was 16% and 8.6%, respectively (Figure 6). Comparing this result with our previous work on intercalated cetirizine into zinc layered hydroxide (ZLH), the latter shows that the inhibition by the free cetirizine is less than the intercalated one [31]. Table 5 shows that CTZAN has higher inhibition compared to CTMAN nanocomposite. This may be due to two factors; the cumulative release of cetirizine from CTZAN is higher than that from CTMAN, and the inhibition by Zn/Al-LDH layer is generally higher than Mg/Al-LDH.

## 3. Experimental Section

### 3.1. Materials

Cetirizine hydrochloric acid (C_21_H_25_ClN_2_O_3_·2HCl, molecular weight 461.5, abbreviated as CT), was purchased from Upha Pharmaceutical Manufacturing (Malaysia) with 99.9% purity and used as received. Other materials including Zn(NO_3_)_2_·6H_2_O, Al(NO_3_)_3_·9H_2_O, Mg(NO_3_)_2_·6H_2_O sodium hydroxide and phosphate-buffered saline solution purchased from Sigma-Aldrich (St Louis, MO) and used as received. Deionized water was used in all the experiments.

### 3.2. Synthesis of the Cetirizine-MgAl and Cetirizine ZnAl Nanocomposite by the Ion-Exchange Method

The pristine Mg/Al-NO_3_ LDH was synthesized by procedure similar to that reported previously [32]. A solution of magnesium with 0.05 mol/L and 0.025 mol/L aluminum nitrates in deionized water was prepared. A solution of sodium hydroxide with 2 mol/L concentrations was added dropwise to Mg/Al solution with vigorous stirring under a nitrogen atmosphere until pH 10.

Cetirizine-Mg/Al-LDH was prepared via. ion exchange process. Fresh Mg/Al-NO_3_ LDH precursor was dropped by a solution of cetirizine (0.2 mol/L) with vigorous stirring, under a nitrogen atmosphere. The pH mixture was 10.0 and aging for 18 h at 70 °C and the precipitate was washed three times and centrifuged. The product was denoted as CTMAN.

The cetirizine-Zn/Al- LDH was prepared via ion exchange process between the nitrate groups in the fresh Zn/Al-NO_3_ LDH with initial molar ratio, 0.1:0.025 mol/L for Zn:Al and the cetirizine anions in the solution (0.3 mol/L), the final pH was 7. The precipitate was aged for 18 h at 70 °C, washed three times and centrifuged. The product was denoted as CTZAN.

### 3.3. Characterization

Powder X-ray diffraction (PXRD) patterns were obtained on a Shimadzu diffractometer, XRD-6000, using CuK_α_ radiation at 30 KV and 30 mA, with a dwell time of 0.5 degrees per minute. FT-IR spectra were obtained using a Thermo Nicolet Nexus FTIR (model Smart Orbit) with 4 cm^−1^ resolution by the standard KBr disc method. Thermogravimetric and differential thermogravimetric analyses (TGA-DTG) were recorded on a Mettler Toledo instrument with a heating rate of 10 °C/min under a nitrogen atmosphere (N_2_ flow rate 50 mL/min). Elemental chemical analysis for Zn, Al and Mg were carried out in an inductively coupled plasma atomic emission spectrometry using a Perkin-Elmer spectrophotometer model Optima 2000DV (Perkin-Elmer) under standard conditions. Carbon, hydrogen and nitrogen were detected in an Elemental Analyzer; a CHNS-932 LECO instrument was used. A Field Emission Scanning Electron Microscope (FESEM; NOVA NANOSEM 230 model) was used to study the surface morphology of the samples. The controlled release study was accomplished using a Perkin Elmer UV-visible Spectrophotometer, Lambda 35.

### 3.4. Release Study of Cetirizine from CTZAN and CTMAN Nanocomposites

Cetirizine release profiles from the nanocomposites were determined at room temperature using phosphate buffered saline solution (PBS) at a concentration of 0.01 mol/L at pH 4.8 and 7.4 [5,13,33]. About 85 mg of each nanocomposite was added to 500 mL of the PBS media. The cumulative amount of cetirizine released into the solution was measured at preset time intervals at *λ*_max_ = 230 nm using a Perkin Elmer UV-Vis spectrophotometer, model Lambda 35.

To compare the release rate of cetirizine from CTZAN and CTMAN nanocomposites, with the physical mixture which contain cetirizine with Zn/Al-LDH or MgAl-LDH. About 0.62 mg of physical mixture was obtained by mixing 0.35 mg cetirizine with the 0.27 mg pristine Zn/Al-LDH to compare the release from CTZAN nanocomposite. In addition, 0.38 mg of cetirizine was mixed with 0.24 mg of Mg/Al-LDH was used to compare with release from CTMAN. The release of the active, cetirizine was determined as described above.

### 3.5. Cytotoxicity Assay

For cytotoxicity study, human Chang liver cells line were seeded into six-well plates when they reached 40%–60% confluence and allowed to incubate overnight at 37 °C under a 5% CO_2_ atmosphere. After incubation for 24 h for cell attachment, the medium in the wells was then replaced with 3 mL of the fresh medium containing CTZAN and CTMAN nanocomposites in a concentration range of 7.8– 1000 μg/mL. A control experiment was performed without treatment by nanocomposites under the same conditions. After 24 h incubation, the effect of nanocomposites on cell viability was determined by trypan blue assay; media was aspirated off, and the cells were harvested by centrifugation then washed with cold phosphate buffered saline (to eliminate the dead cells). Then, 10 μL of cells was mixed with equal volume of 0.4% trypan blue (Sigma, USA) and was counted by using Neubauer haemocytometer (Weber, England) by clear field microscopy (Nikon, Japan). Only viable cells were counted. Each compound and control was assayed in triplicate.

### 3.6. Cell Culture Conditions and Anti IgE-Induced Histamine Release

Rat basophilic leukemia cells RBL-2H3 are obtained from American Type Tissue Collection (ATCC; Rockville, MD). These cells are grown in Dulbecco’s Modified Eagle Medium (DMEM), which supplemented with 10% Fetal Bovine serum (FBS). The media contain penicillin (100 U/mL) and streptomycin (100 μg/mL). Cells were grown at 37 °C in a humidified 5% CO_2_ incubator.

For the determination of histamine release, RBL-2H3 cells were plated at 1 × 10^6^ cells/well by addition 0.5 mL of a 2 × 10^6^ cells/mL suspension to each well of a 24-well and incubated for 18 h to assure attachment and 90% confluence. After the media was aspirated off, IgE (0.2 μg/mL, MP Biomedicals) was added in DMEM. Cells were incubated at 37 °C for 1 h. After that, each well was washed with release buffer containing 1 mM CaCl_2_, 40 mM NaOH, 0.1% BSA 119 mM NaCl, 5 mM KCl, 5.6 mM glucose, 25 mM PIPES and 0.4 mM MgCl_2_. The release buffer containing Anti-IgE (1.25 μg/mL) and different concentration of cetirizine, CTZAN, CTMAN, Zn/Al-LDH and Mg/Al-LDH was then added to each well and cells were incubated at 37 °C for 10 min. The amount of histamine release was determined using a histamine RIA kit (MP Biomedicals).

## 4. Conclusions

The intercalation of cetirizine into two types of layered double hydroxides, namely Zn/Al LDH and Mg/Al LDH, using ion exchange method to obtain CTZAN and CTMAN nanocomposites, was successfully accomplished. The XRD results confirmed the intercalation process in which cetirizine is arranged between the interlayers as tilted bilayers. The intercalated amount of cetirizine into the CTZAN and CTMAN was estimated to be 57.2% and 60.7%, respectively. FTIR study shows that the bands of the resulting nanocomposites correspond to the characteristic functional groups of the cetirizine and LDH structures, which support the intercalation episode. Interestingly, the release rate of cetirizine from nanocomposites depends on the acidity of the media and type of host, where the release rate at pH 7.4 is remarkably lower than that at pH 4.8, and the release of cetirizine from CTMAN is lower than CTZAN. The inhibition of histamine by free cetirizine is higher than the cetirizine-intercalated into CTZAN and CTMAN, in agreement with the sustained release properties of both nanocomposites. The study showed no cytotoxicity effect for CTZAN and CTMAN nanocomposites up to 1000 μg/mL

## Figures and Tables

**Table 1:** XRD data of diffraction peaks and the lattice parameters of CTZAN and CTMAN nanocomposites.

Samples	*d*_003_ (Å)	*d*_006_ (Å)	*d*_009_ (Å)	*d*_012_ (Å)	*d*_110_ (Å)	Average *d* value	*a* (Å)	*c* (Å)	M^2+^/Al^3+^	*x*
**CTZAN**	31.90	15.93	10.64	7.88	1.51	31.8	3.02	95.5	3.3	0.23
**CTMAN**	31.15	15.33	10.15	7.66	1.51	30.8	3.02	92.4	2.03	0.33

**Table 2:** Fourier transform infrared assignment for cetirizine, CTZAN and CTMAN [23,24].

Assignments	Cetirizine	CTZAN	CTMAN
*v* (O–H)	3432 for O–H in carboxylic group	3445 in the layer; H_2_O	3442
*v* (CH_2_)	2984–2949	2958–2817	2958–2819
*v* (COOH)	1740	-	-
*v* (ϕ. Para- subst.)	1601	1601	1589
*v* (ϕ. mono- subst.)	1496, 1077 and 758	1487	1488
*v* (C–Cl)	1457	1452	1453
*v* (C–O) in carboxylic group	1435, 1383, 1356 and 1319	-	-
2 adj. ϕ	846 and 809	853 and 805	853 and 805
*v* (CH mono- subst.)	758	758	758
M-O	-	428	446
*v*_as_ (COO^−^)	-	1601	1589
*v*_s_ (COO^−^)	-	1408	1408

**Table 3:** Elemental chemical compositional and empirical formula for cetirizine nanocomposites.

Samples	M^2+^% a	Al^3+^% a	C% b	N% b	C/N	Drug% b	Empirical formula
**CTZAN**	21.5	2.7	37.2	4.2	8.8	57.2	[Zn_0.77_Al_0.23_(OH)_2_](CT)_0.22_(NO_3_^−^)_0.01_.1.16H_2_O
**CTMAN**	8.03	4.37	39.4	4.4	9.04	60.7	[Mg_0.67_Al_0.33_(OH)_2_](CT)_0.33_.1.4H_2_O

M^2+^ is Zn for CTZAN and Mg for CTMAN,

a calculated upon ICP data;

b calculated upon CHNS data.

**Table 4:** Correlation coefficient (*R*^2^), rate constants (*k*) and half time (*t*_1/2_) obtained by fitting the cetirizine release data from CTZAN and CTMAN nanocomposites into solutions at pH 4.8 and 7.4.

Samples	pH	Saturation release (%)	*R*^2^			Pseudo-second order	

			Pseudo-first order	Pseudo-second order	Parabolic diffusion model	Rate constant *k* (mg/min)	*t*_1/2_ (min)
**CTZAN**	7.4	95.6	0.9644	0.9885	0.8811	1.07 × 10^−4^	84.3
**CTZAN**	4.8	96	0.9190	0.9985	0.8145	3.24 × 10^−4^	30.4
**CTMAN**	7.4	96.3	0.9369	0.9983	0.7781	4.14 × 10^−5^	233.3
**CTMAN**	4.8	97.8	0.8056	0.9914	0.7106	1.65 × 10^−4^	56.8

**Table 5:** Inhibition percentage of histamine release in RBL2H3 cells by the cetirizine, CTZAN, CTMAN, Zn/Al-LDH and Mg/Al-LDH.

Concentration (ng/mL)	Inhibition (%)				

	Cetirizine	CTZAN	CTMAN	Zn/Al-LDH	Mg/Al-LDH
1000	80.0	73.5	45.6	3.7	2.6
500	73.7	64.6	40.3	3.7	2.0
250	58.1	44.0	31.7	1.4	1.5
125	50.2	33.9	26.4	1.8	0.2
62.5	29.0	20.1	14.2	0.7	0.2
31.25	19.3	12.6	6.1	−1.2	0.1
15.63	8.5	4.3	1.6	1.7	−2.9
